# Psychological distress and associated factors among asthmatic patients in Southern, Ethiopia, 2021

**DOI:** 10.1186/s40733-023-00093-z

**Published:** 2023-06-05

**Authors:** Kidist Ashager, Mulualem Gete Feleke, Sindu Degefu, Eshetu Elfios, Asmamaw Getnet, Elias Ezo, Mezinew Sintayehu

**Affiliations:** 1 Department of Nursing, School of Nursing, College of Medicine and Health Sciences, Wolaita Sodo University, Sodo, Ethiopia; 2 Department of Nursing, College of Medicine and Health Sciences, Debre Markos University, Debre Markos, Ethiopia; 3 Department of Nursing, College of Medicine and Health Sciences, Wachamo University, Hosaena, Ethiopia

**Keywords:** Psychological distress, Asthma patients, Ethiopia

## Abstract

**Background:**

There is an increased prevalence of psychological distress in adults with asthma. Psychological distress describes unpleasant feelings or emotions that impact the level of functioning. It is a significant exacerbating factor in asthma control. Addressing factors that contribute to psychological distress in those asthma patients improves asthma outcomes. So, this study aimed to assess the prevalence of psychological distress and associated factors among asthmatic patients at Hawassa public hospitals, Ethiopia, 2021.

**Methods:**

Institution-based cross-sectional study design was used to select 394 asthma patients. Proportional allocation and systematic sampling techniques were used to select study participants. A logistic regression model was used to assess the predictors and psychological distress of the asthmatic patient. The association was interpreted using the odds ratio and 95% confidence interval.

**Result:**

A total of 394 asthma patients participated in the study, giving a response rate of 93.4%. The prevalence of psychological distress among asthmatic patients was 51% [95%CI: 46%-56%]. Participants who had comorbid medical illness [AOR: 6.049, 95% CI (3.131–11.684)], experienced stigma [AOR: 3.587, 95%CI (1.914–6.723)], chewed khat [AOR: 7.268, 95%CI (3.468–15.231)], had poor social support and had uncontrolled asthma were significantly associated with psychological distress in asthmatic patients.

**Conclusion:**

This study demonstrated that the prevalence of psychological distress was found to be high among asthmatic patients. Social support, stigma, chewing khat, comorbid medical illness, and poor asthmatic control had significantly associated with psychological distress in asthmatic patients.

## Background

Asthma is a lifelong respiratory disease characterized by airway obstruction, airway inflammation, and bronchial hyperresponsiveness that produce recurrent episodes of wheezing, coughing, and dyspnea [1]. Patients with asthma due to their respiratory condition are at increased risk of psychological distress, so it is important to focus on psychological elements in the management of psychological distress [2].

Psychological distress (PD) had both emotional and physiological manifestations that interfere with activities of daily living. It is characterized by symptoms of depression, anxiety, and somatic symptoms [3, 4]. Evidence showed that asthmatic patients had a greater psychological burden than other patients [4, 5]. Psychological distress can alter multiple physiological systems including emotional perceptions, neuroendocrine pathways, and immune networks to produce either exhaustion, dysregulation, or both [6, 7].

Globally, WHO estimates that 300 million people were suffering from asthma, with approximately 250,000 deaths annually [8]. The Global Initiative for Asthma (GINA) estimates that the global prevalence of asthma ranges from 1 to 18% of the total population of different countries [8, 9]. Studies in Ethiopia showed that the prevalence rate of asthma ranged from 4.9% to 26.6% [10, 11].

Psychological factors seem to have a major role in asthma; thus, including psychological assessment in patients’ treatment is quite reasonable [12]. Psychological distress is a set of painful, mental, and physical symptoms that are associated with normal fluctuations of mood in most people [13, 14]. In some cases, however, psychological distress may indicate the development of a major depressive disorder, anxiety disorder, schizophrenia, somatization disorder, or a variety of other clinical conditions. It has been implicated as potentially contributing to asthma severity [12]. PD may be a risk factor for asthma-related morbidity and mortality also it has the potential to affect asthma at multiple levels, directly inducing exacerbations in some patients and increasing the incidence and severity of asthmatic responses. PD has also an impact on hospitalization, prolonged medication use, and the prognosis of asthma [15, 16].

In Canada, the ratio of psychological distress among people with asthma as compared to healthy individuals ranged from 1.1 to 2.7 [17]. Another study in Kuwait showed that 69% of asthmatic patients had psychological distress [18]. In Africa, Asthma is a significant and major public health problem that is not given enough emphasis and attention [15, 19]. The burden of asthma can be reduced by treating PD and establishing asthma control, hence it is much more pertinent to assess PD prevalence and risk factors when calculating the burden of asthma because the resulting estimate can be viewed as a "preventable" burden [20, 21].

The body of evidence shows that psychological distress has an impact on asthma patients daily lives, subjective interpretation of symptoms, and adherence to treatment [22]; Moreover, the presence of the disease itself may have an impact on patients’ affective sphere by representing an obstacle to optimal disease management. Some people were forced to change their occupations due to poor work attendance caused by illness or the inability to perform certain tasks. They were also burdened by additional household and financial responsibilities. When they miss work, it results in increased use of healthcare resources and loss of productivity [23, 24].

The presence of psychological morbidity is, however, frequently unrecognized or not assessed by the usual clinicians treating asthma patients. So far, there is no data on the prevalence of physiological distress among asthmatic patients and its associated factors in Ethiopia. Hence, this study aimed to assess the prevalence of psychological distress and associated factors among asthma patients attending public hospitals located in Hawassa, Ethiopia. Identifying and controlling factors like psychological distress aids or improves patients’ control of their asthma over and above pharmacotherapy and helps to reduce the burden of asthma.

## Methodology

### Study area, design, and period

A cross-sectional study was conducted from March 20 to April 20/2021 at Hawassa University Comprehensive Specialized Hospital and Adere General Hospital, Hawassa, SNNPR Ethiopia. Hawassa is the regional city of SNNPR located 273 km from the capital city of Ethiopia, Addis Ababa. The two public hospitals, namely HUCSH and HAGH provide both outpatient and inpatient health care services to about 15 million and 350,000 people respectively since their opening in 2004.

### Source and study population

All asthma patients on follow-up at Hawassa University comprehensive specialized hospital and Adare general hospital were the source population.

### Inclusion and exclusion criteria

All adult asthmatic patients whose ages were ≥ 18 years and who had a follow-up in HCSH and Adare Hospital were included. Patients in respiratory distress who were not able to respond due to severe illness were excluded.

### Sample size determination and sampling procedure

The sample size was determined by using a single population proportion formula by considering the following assumptions: prevalence of PD among asthma = 50% (0.5) because there is no previous study done on this topic in Ethiopia, the value of Zα/2 = 1.96 which is Z score of 95% confidence interval, and margin of error = 5% (0.05).This yields an initial sample size of 384. By considering adjustment for the expected non-response rate (10%), the final calculated sample size was 422.

The two public hospitals had asthmatic chronic follow-up units that were conveniently selected. During the data collection period at each chronic follow-up outpatient department (OPD), 600 and 285 patients were attended at HUCSH and Adare hospital respectively. The samples were proportionally allocated to each hospital. A Systematic sampling techniques was used to select study participants. After the first respondent drown by lottery method then every two intervals was interviewed till the sample size was reached.

### Variables of the study

Psychological distress of the asthmatic patient was a dependent variable.

Socio-demographic characteristics (sex, age, marital status, residence, educational status, employment status, family history of asthma, and distance from the hospital); clinical factors (level of asthma symptom control, adherence to medications, comorbidity, and history of past mental illness); behavioral factors (social support, stigma, and substance use) were independent variables.

### Operational definition

#### Level of asthma symptom control

Based on the Global Initiative for Asthma (GINA) guidelines, as follows: symptoms within 4 weeks, none of the symptoms were considered controlled, 1–2 symptoms were considered partly controlled, and 3–4 symptoms were considered uncontrolled. This tool uses frequency of symptoms, night waking due to asthma, limitation of activity, and frequency of reliever medication use [25]. This tool has been correlated to other standardized asthma control scores [26, 27].

#### Social support

Is the support gained from family and non-family members. The score ranges from 3 to 14, with high values representing strong levels of social support and low values representing poor levels of social support. The Oslo social support scale (OSSS)-3 score can be operationalized into three broad categories of social support. 3–8 poor social support, 9–11 moderate social support, and 12–14 strong social support [28].

#### Psychological distress scale (K-10)

The item score sum ranges from 10 to 50, which indicates an increasing degree of psychological distress. The score ranges from 10–19 likely to be well, 20–24 likely to have a mild disorder, 25- 29 likely to have a moderate disorder, and 30- 50 likely to have a severe disorder. Respondents had psychological distress when their score on the scale were 22 or above [29]. Each item on the K-10 experienced by a patient was recorded using a five-point Likert scale with responses ranging from ‘none of the time’ to ‘all of the time’.

#### Medication adherence

Respondents who scored the sum of 8 had high adherence, those who scored 6–8 had medium adherence, and those who scored < 6 had low adherence to their medication [30].

#### Stigma

The SSCI-8 scores items on a 5-point Likert scale from none of the time (score 1) to all of the time (score 5). The total score ranges from 8 to 40, a higher score indicates higher levels of perceived stigma. Those who scored above the mean value [12] were considered to have a stigma [31].

#### Current substance use

Refers to the use of alcohol, khat, and cigarette for the past one month [32].

### Data collection tool and procedures

Data were collected by using face-to-face interviews at chronic follow-up units. Structured questionnaires were used to acquire demographic information, behavioral factors, clinical related data and psychological distress. The questionnaire was developed based on the existing literature [25, 28–33].

The questionnaire had four sections socio-demographic characteristics, clinical related factors, personal related factors, psychological distress the Kessler 10-item (K-10) scale was used [29]. Personal factors had 25 questions; in its sub-sections, social support was assessed using Oslo 3-item social support scale [28]. Regarding substance use participants who smoked cigarettes within a month were considered smoker, participants who drank alcohol within a month were considered users of alcohol; participants who chewed khat within a month were considered users of khat.

Stigma was also assessed using SSCI- 8 items which assess enacted and internalized stigma and have adequate internal reliability and validity in relation to PD [31].

Four BSc nurses for data collector and one psychiatry nurse for supervisor were recruited during the data collection period (both the data collectors and the supervisor were not the same hospital).

### Data quality assurance

Pre-test was done on 5% of the sample size before the data collection at Worabe comprehensive specialized hospital and some modifications were made to the clarity of questions and wording. The training was given to data collectors and a supervisor. All the questions were prepared in English and translated into the Amharic by an expert who was fluent in both languages and back-translated to English to see its consistency. On every other day, the supervisor checked the data for completeness.

### Data processing and analysis

Data were entered, coded, and edited into EPI-data version 3.1 and exported to SPSS version 25 for analysis. Descriptive statistics were used to illustrate the means, standard deviations, medians, and frequencies of the study variables. Bivariate analysis was computed, and those variables whose *p*-values ≤ 0.2 were fitted into the backward stepwise multivariate logistic regression model. The Hosmer and Lemeshow goodness of fit test for the model was checked. Finally, the degree of association was interpreted by using an odds ratio with a 95% confidence interval. The *p*-value ≤ 0.05 was considered statistically significant.

## Result

### Socio-demographic characteristics

Of the total of 422 asthmatic individuals invited, 394 participated in our study with a response rate of 93.4%. Of these 207(52.5%) were females. The majority of the participants, 323(82%) lived in the urban area. Most of the participants were employed (governmental or non-governmental) 91(24.2%) and 146(37.1%) respondents were married (Table 1).

**Table 1:** Socio-demographic characteristics of asthmatic patients Hawassa, southern Ethiopia, 2021 (*n* = 394)

Variables	Categories	Frequency	Percent
Sex	Male	187	47.5
	Female	207	52.5
Age	18–30	120	30.5
	31–45	124	31.5
	46–60	97	24.6
	61–75	46	11.7
	76 and above	7	1.8
	Mean ± SD	40.5 ± 12.85	
Residence	urban	323	82
	rural	71	18
Educational status	Unable to read and write	74	18.8
	Elementary (1–8)	74	18.8
	High school (9–12)	109	27.7
	Diploma &above	137	34.8
Marital status	married	146	37.1
	single	116	29.45
	widowed	88	22.3
	divorced	44	11.2
Occupation	Government employee	98	24.9
	daily laborer	8	2
	Student	46	11.7
	Retired	53	13.5
	Self-employed	100	25.4
	Farmer	41	10.4
	Unemployed	48	12.2
Distance of home from hospital	100 m-5 km	282	71.6
	6 km-10 km	42	10.7
	11 km-20 km	34	8.6
	20 km-30 km	36	9.1

### Prevalence of psychological distress among asthmatic patient

**Fig. 1:**
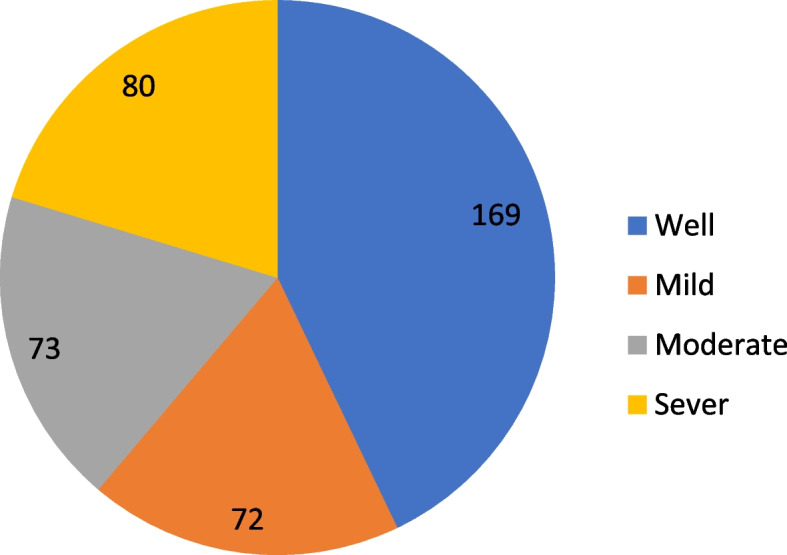
Level of psychological distress among asthmatic patients at Hawassa public hospital, Ethiopia, 2021

Among a total of 394 asthmatic patients, more than half (51%) experienced psychological distress (Fig. 1).

### Clinical factors

Out of the total of 394 respondents, 273 (69%) had uncontrolled asthmatic symptoms. Of the total number of respondents, 38 (9.6%) had high adherence to their medication (Table 2).

**Table 2:** Descriptive statics of clinical factor result of asthmatic patients Hawassa, southern Ethiopia, 2021

Variables	Categories	Frequency	Percent
Had past history of mental illness	Yes	52	13.2
Kind of past mental illnesses	Depression	16	30.8
	Anxiety	23	44.2
	Schizophrenia	6	11.5
	Others (PTSD, Eating disorder, mood disorder)	7	13.5
Family history of mental illness	Yes	89	22.6
Family history of asthma	Yes	222	56.3
Comorbidity	Yes	144	36.5
Kind of comorbidity	Diabetes	45	11.4
	Cardiac disease	53	13.5
	Cancer	4	1
	Arthritis	21	5.3
	Hypertension	21	5.3
Duration since diagnosis of asthma	1-5 yr	94	23.9
	6-10 year	96	24.4
	11-15 year	51	12.9
	16-20 year	67	17
	21 year and above	86	21.8
Asthmatic control level	Uncontrolled	273	69.3
	Partly controlled	91	23.1
	Controlled	30	7.6
Medication adherence	high	38	9.6
	Medium	194	49.2
	Low	162	41.1

### Behavioral related factors

Out of 394 study participants, 215 (54.6%) of them drank alcohol. In terms of smoking status, 30(7.6%) were smokers. Regarding khat, 89 (22.6%) of them chewed khat. Regarding stigma, 146 (37.1%) of asthmatic patients experienced stigma. One hundred four (34.01%) of the participants had poor social support (Table 3).

**Table 3:** Patient related factors in asthmatic patients in Hawassa, southern Ethiopia, 2021

Variables	Categories	Frequency	Percent
Social support	Poor	134	34
	Moderate	128	32.5
	Strong	132	33.5
Stigma	Yes	146	37.1
Khat Chewing	Yes	89	22.6
Alcohol drinking	Yes	215	54.5
Smoking cigaratte	Yes	30	7.6

### Factors associated with psychological distress among asthmatic patients

After adjusting potential confounders of the covariates, comorbid medical diseases, asthma symptom control level, social support, stigma, and khat chewing were significantly associated with psychological distress among asthmatic patients. Respondents who had co-morbid medical diseases were 6 times [AOR: 6.049, 95%CI (3.131–11.684)] more likely to develop PD than those who didn’t had co-morbid medical diseases. Respondents who had controlled their symptoms were 77% less likely to develop PD than those who had uncontrolled asthma [AOR: 0.229, 95%CI (0.066–0.797)]. Those who experienced stigma were 3.6 times more likely to develop PD than those who were not stigmatized [AOR: 3.587, 95%CI (1.914–6.723)]. Those respondents who had strong social support were 89% [AOR: 0.111, 95%CI (0.050–0.246) less likely to develop PD than those with poor social support. Those asthma patients who chewed khat were 7 times more likely [AOR: 7.268, 95%CI (3.468–15.231)] to develop PD than those who didn’t chew khat (Table 4).

**Table 4:** Bivariable and Multivariable Logistic regression analysis model for factors associated with Psychological Distress among asthma patients in Hawassa, southern Ethiopia, 2021(*n* = 394)

**Variables**	**Categories**	PD		**COR (95%CI)**	**AOR(95%CI)**	***P*-value**
		Yes				
		Frequency	Percent (%)			
Residence	urban	161	40.9	1	1	
	rural	43	10.9	1.55(0.67–5.78)	1.82(0.34–3.67)	0.45
Comorbid	No	91	23	1	1	
	Yes	110	27.9	5.653(3.558–8.981)	6.049 (3.131–11.684)**	0.001
Asthma symptom control level	Uncontrolled	173	43.9	1	1	
	Partly controlled	23	5.8	0.191(0.112–0.325)	0.197(0.098–0.397)**	0.000
	Controlled	5	1.2	0.114(0.042–0.308)	0.229(0.066–0.797)*	0.020
Educational status	Unable to read and write	41	10.4	1	1	0.240
	Elementary (1–8)	37	9.3	0.8(0.05–6.67)	0.56(0.45–3.67)	0.824
	High school (9–12)	55	14	0.82(0.06–4.88)	0.67(0.05–6.67)	0.501
	Diploma &above	68	17.3	0.79(0.24–4.55)	0.89(0.05–6.67)	0.786
Social support	Poor	106	26.9	1	1	
	Moderate	62	15.7	0.248(0.144–0.427)	0.269(0.129–0.562)*	0.001
	Strong	33	8.4	0.088(0.050–0.156)	0.111(0.050–0.246) **	0.000
Stigma	No	90	22.8	1	1	
	Yes	111	28	5.568(3.516–8.817)	3.587 (1.914–6.723) **	0.000
khat Chewing	No	132	33.5	1	1	
	Yes	69	17.5	4.522(2.617–7.812)	7.268(3.468–15.231)**	0.002

NB: “**” = *p* < 0.001: strongly significant association; “*” = *p* < 0.05: statistically associated; “1” = reference group

## Discussion

This study aimed to assess the prevalence of PD in asthma patients and pinpoint the factors associated with psychological distress that occur in asthmatic patients. PD is considered to be a common presentation in asthma patients. Psychological distress can alter multiple physiological systems including emotional perceptions, neuroendocrine pathways, and immune networks.

In this study, the prevalence of PD among asthma patients was 51%. This finding is higher than studies conducted in US 7.5% [34], Australia 17.9% [35], and Jordan 28% [36]. These discrepancies could be due to variations in socio-demographic differences among the study areas, in the level of knowledge among study populations, the health service delivery system in the study areas, and the data collection method they used.

However, this finding is lower than in studies conducted in Kuwait (69%) [18], and in Iran (64.7%) [5]. These differences could be attributable to several factors, including the study design, and the diagnostic tools they used.

This study revealed that the level of asthma symptom control was significantly associated with PD. Respondents who had controlled their symptoms were 77% less likely to develop PD than those who had uncontrolled asthma [AOR: 0.229, 95%CI (0.066–0.797). This finding is supported by studies done in Egypt [12] and Canada [17]. This may be due to the fact that poor asthma symptom control is associated with current smoking, history of exacerbations, and impaired lung function and leads to psychological distress.

Respondents who had comorbid medical diseases were 6 times more likely to develop PD than those who didn’t have any comorbid medical diseases; this is in line with another study done in US National Health Interview Survey [34]. This could be because patients with co-morbidity are often on complex medication regimens.

In this study, respondents who had strong social support were 88%less likely to develop PD than those who had poor social support; this idea is supported by a study done in Jordan [36]. This might be due to the social support that can improve emotional well-being (receiving love and empathy) and practical help (gifts of money, family commitments to prepare and buy their medication, and care assistance).

In the current study, those participants who chewed khat were significantly associated with PD, which is supported by the US National Health Interview Survey report [34]. Khat use was associated with anxiety, and a higher rate of symptoms of depression, anxiety, and stress.

The present study showed that stigma 146(37.1%) of asthmatic patients were a significant associated with developing PD. This is supported by the descriptive study in Jordan 72.8% of participants feel sometimes stress due to people's influence and misunderstanding of the disease and about 68% of them believed that people sometimes can do nothing for them [36].

## Limitation of the study

Due to the cross-sectional design of this study, it is not possible to draw any conclusions regarding causality. The interviewer assessed the patient's substance use, and as result, the patient may be afraid to answer the truth.

## Conclusion

Generally, in this study, more than half of the participants had psychological distress. This study also identified that factors like social support, stigma, chewing khat, comorbid medical illnesses, and asthma symptom control level were significantly associated with PD in asthmatic patients.

## Recommendations

Attention should be given to factors that cause psychological distress in asthmatic patients by giving clear and honest information on the triggering factors of their disease and medication adherence. There should be designed programs to give training to healthcare personnel to increase their clinical acumen at recognizing PD. It is better for future researchers to undergo longitudinal research on risk factors for PD and to study other additional variables that may cause PD.

## Data Availability

All data are available in the manuscript.

## Abbreviations

AOR: Adjusted Odds Ratio
AGH: Adare General Hospital
CI: Confidence Interval
COR: Crud Odds Ratio
HUCSH: Hawassa University Comprehensive Specialized Hospital
PD: Psychological Distress
SSCI: Stigma Scale for Chronic Illness
SNNPR: Southern Nation and Nationalities People’s Region
SPD: Sever Psychological Distress
WHO: World Health Organization
